# Mucormycosis in a case of multisystem inflammatory syndrome in children: “double trouble” (case report)

**DOI:** 10.11604/pamj.2022.43.84.33635

**Published:** 2022-10-18

**Authors:** Keta Vagha, Revat Meshram, Sham Lohiya, Spandana Madirala, Chitturi Venkata Sai Akhil, Patel Zeeshan Jameel, Jayant Vagha

**Affiliations:** 1 Department of Pediatrics, Jawaharlal Nehru Medical College, Sawangi (Meghe), Wardha, Maharashtra, India

**Keywords:** COVID-19, multisystem inflammatory syndrome, SARS-CoV-2, mucormycosis, pediatric, case report

## Abstract

Multisystem inflammatory syndrome (MIS-C) in the pediatric age group is a clinical syndrome in children and adolescents which is recognized in association with a high local prevalence of Corona Virus Disease-2019 (COVID-19). Mucormycosis is a severe form of fungal infection and often affects immunocompromised patients. It is associated with high morbidity and mortality and is characterized by extensive angioinvasion and necrosis of the affected tissue. Currently, this dreaded mucormycosis is rising among COVID-19 pediatric patients during their treatment period or after their discharge from the hospital. It is also called COVID-19- associated mucormycosis (CAM) or black fungus. In the head and neck area, rhino-orbital-cerebral mucormycosis is the most common presentation and a fatal clinical entity associated with COVID-19 infections. There are several cases of mucormycosis reported in cases with COVID-19 infection, but there is limited data available for the development of mucormycosis in MIS-C. Here, we report a case of a nine years old girl who developed mucormycosis while suffering from MIS-C. The patient was brought to our institute with complaints of fever for 3 days associated with redness of the eyes and swelling behind both ears and bilateral conjunctival congestion. Subsequently, she started showing signs of end organ damage in form oliguria and deranged liver function. Her COVID-19 antibody titer was positive and hence was diagnosed as MIS-C. She had prolonged hospitalization during which she started developing black discoloration over the nose. The histopathology report of the lesion was suggestive of mucormycosis. Eventually, the patient died due to multiple organ dysfunction. There is not only an association of mucormycosis in COVID-19- positive patients, but it shows some association with its complications like MIS-C. There is very limited data available for the association of mucormycosis and MIS-C but early diagnosis and intervention play a vital role in the outcome of the patient.

## Introduction

COVID-19 infection is a highly contagious and rampantly spreading disease of the respiratory tract caused by severe acute respiratory syndrome coronavirus 2 (SARS-CoV-2). Initial reports showed that, it presented in a mild form in children but with the progression in the viral spread, the severity of the disease is increasing in children. Along with the increasing severity of COVID-19 in children, a new multisystem disorder has been associated with children who have had been infected with COVID-19 in the past, [1]. It is characterized by persistent fever spikes, usually rash, increased inflammatory markers and also laboratory evidence of multiorgan dysfunction. In some cases, it also shows features of Kawasaki syndrome like rash, conjunctival and mucosal injection, swelling of hands and feet, and coronary artery dilation and in a few other cases, it shows features of toxic shock syndrome like erythroderma, renal involvement, and hypotension [2]. Recent reports showed that there was an increasing association between COVID-19 infection and the black fungus infection called mucormycosis which has been proven to be fatal. It is spreading rapidly and is considered to affect all age groups suffering from COVID-19 infection. Mucormycosis is linked with increased mortality and it can be confined to the local tissue or have a systemic invasion. Mucormycosis is the third most common fungal infection preceded by candidiasis and aspergillosis [3]. The patients with MIS-C require mechanical ventilation, and have longer hospital stays and those who are on systemic steroids for a longer duration are predisposed to develop mucormycosis [4]. In the pediatric age group, MIS-C is itself a post-COVID-19 complication. Patients with MIS-C usually have varied presentations. But in most cases, it is found to have increased inflammatory markers and strong positive SARS-CoV-2 antibody titers. Many studies showed a strong positive association between COVID-19 infection and mucormycosis. However, there is no literature available for an association of MIS-C and mucormycosis. Here we report a patient who presented with MIS-C and later developed mucormycosis. As there is limited literature on this, we review the literature surrounding the occurrence of mucormycosis after COVID-19 and also the occurrence of MIS-C post-COVID-19.

## Patient and observation

**Information of the patient:** a nine-year-old female was brought to our institute with complaints of high grade fever for three days and redness of eyes for 2 days.

**Clinical findings:** on examination, her heart rate was 112 beats/min, respiratory rate- 34 breaths/min, blood pressure- 82/50 mmHg in right arm supine position and SpO_2_- 94% on room air. She also had submandibular and postauricular lymphadenopathy with no systemic abnormality.

**Diagnostic assessment:** her baseline blood investigations were sent which showed mildly elevated white blood cell count (12500/cumm) and deranged liver function test (AST-99 IU/L) with mildly elevated Urea (53 mg/dL) and normal creatinine (0.7mg/dL). Considering the diagnosis of MIS-C, her inflammatory panel consisting of C reactive protein (CRP), serum ferritin, D-Dimer levels with interleukin- 6 along with COVID-19 antibody levels were sent on 2^nd^ day of admission. The inflammatory panel was highly elevated i.e. CRP- 27 mg/dL, ferritin >1000 ng/mL, interleukin-6 -16.5 pg/ml, D-Dimer level- 8.1 ng/ml. The titer of COVID-19 antibody was also positive (10.9).

**Timeline of current episode:** she was started intravenous isotonic fluids and antibiotics therapy. Gradually, her blood pressure started falling further and it went below the 5^th^ centile for her age and height for which she required ionotropic support. She had persistent fever spikes. Patient had gallop rhythm on auscultation. A 2-D echo was done which was suggestive of left ventricular dysfunction with an ejection fraction of 37% which helped us to differentiate it from Kawasaki Disease as there was no involvement of coronaries.

**Diagnosis:** according to World Health Organization (WHO) [5] she was fulfilling the case definition of MIS-C- as she was 9 years old, with a fever of more than or equal to three days and conjunctivitis with hypotension and cardiac involvement and coagulopathy and most importantly the antibody titer against COVID-19 being strongly positive.

**Therapeutic intervention:** therefore, keeping the diagnosis of MIS-C in mind she was started on systemic steroids and Enoxaparin. Subsequently, her urine output started to decrease suggestive of intrinsic renal which was reflected in the sequential abnormal kidney function tests. Following the protocol to manage MIS-C, she was given intravenous immunoglobulin at 2 gms/kg.

**Figure 1:**
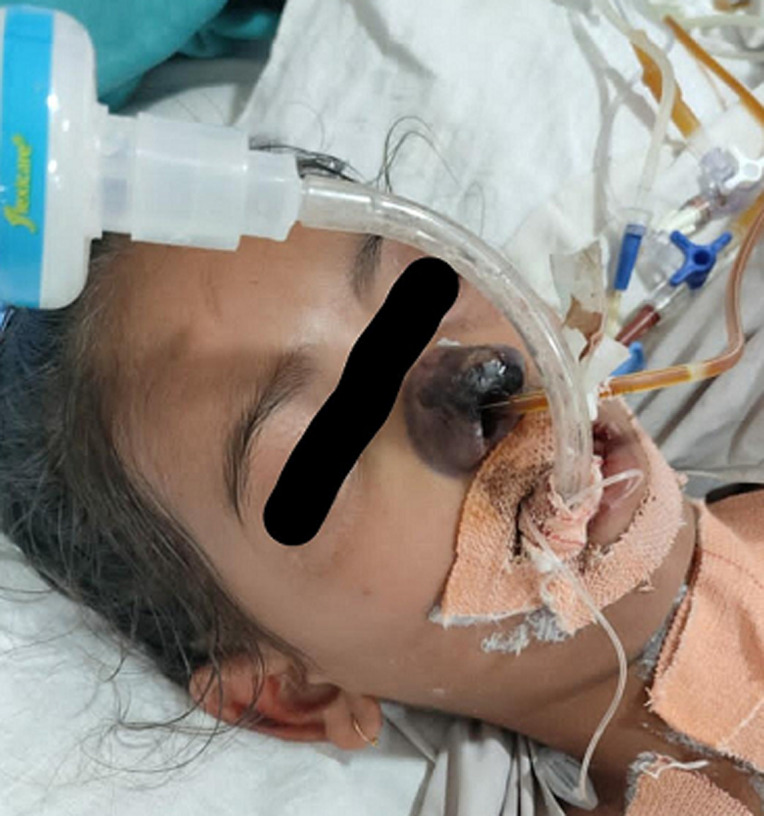
mucormycotic (black coloured) changes on the nose

**Figure 2:**
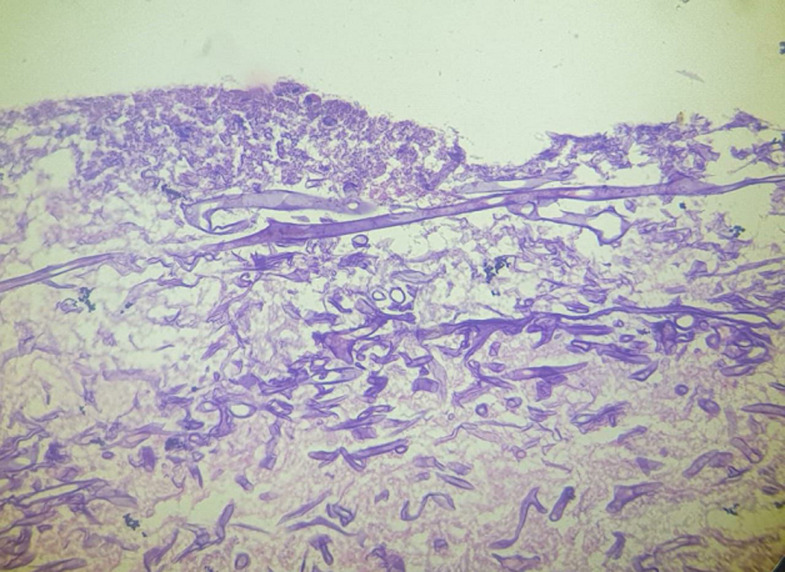
section stained with haematoxylin and eosin, in a high power field (400x magnification) showing longitudinal and transverse sections of thick walled a septate hyphae which show branching at right angles surrounded by inflammatory infiltrate and necrotic debri, suggesting a mucormycotic pathology on histopathology

**Follow-up and outcome of intervention:** patient had episodes of hyperglycemia for which insulin boluses were given. The patient still had intermittent fever spikes for which the antibiotics were upgraded. The blood culture was sterile. The sequential blood investigations started showing progressive thrombocytopenia and anemia along with further elevated inflammatory markers. Because of the increasing respiratory distress and falling oxygen saturation, she was put on mechanical ventilation. There was no hemodynamic improvement in the patient. Her progressive end organ damage was evident with deteriorating kidney function tests and liver function tests. On the 7^th^ day of admission, a black-coloured lesion was found on the nose beside the insertion of ryles tube (Figure 1). Considering it as pressure necrosis, the tube was removed, but the black lesion was spreading in the adjacent nasal area. A sample was taken from the area and sent for histopathological assessment which confirmed the diagnosis of mucormycosis (Figure 2). She was started on injectable antifungal. But the patient showed progressive hemodynamic and multiorgan dysfunction because of which the patient eventually died despite all the resuscitative measures.

**Ethics approval and consent to participate:** the authors certify that they have obtained all appropriate patient consent forms. In the form the patient's parents have given their consent for patient's images and other clinical information to be reported in the journal. The patient's parents understand that their names and initials will not be published and due efforts will be made to conceal their identity, but anonymity cannot be guaranteed.

**Consent for publication:** written informed consent was obtained from the patient's parents for publication of this case report and any accompanying images and videos. A copy of the written consent is available for review by the Editor-in-Chief of this journal.

## Discussion

**Figure 3:**
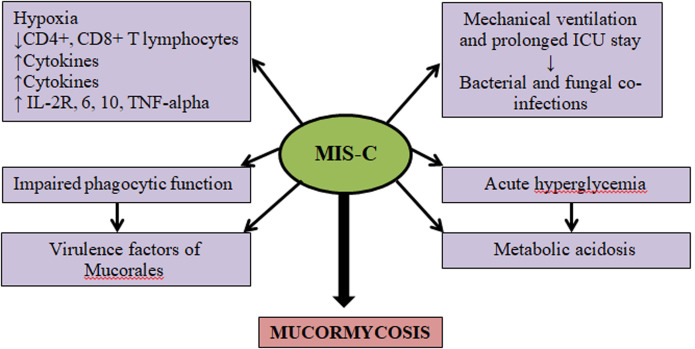
postulated pathophysiology of mucormycosis in multisystem inflammatory syndrome in children

The severe acute respiratory syndrome caused by COVID-19 virus has shown complications due to secondary invasion of opportunistic bacterial and fungal infection. A patient with MIS-C provides a favorable environment of decreased oxygen saturation (hypoxia), elevated plasma glucose (stress-induced hyperglycemia, steroid-induced hyperglycemia), high iron level (increased ferritin level due to inflammation) and acidic medium (metabolic acidosis) for the mucorale spores to germinate [6]. Mucor spores can even be present in the nasal mucosa of a non-sick individual but because of its low disease-causing capacity, it may not manifest. If the patient becomes immunosuppressed, like in MIS-C due to prolonged steroid therapy or other mentioned reasons, the fungus with low disease causing capacity may manifest in such individuals. Rhinocerebral presentation is the most common form [7]. Mucormycosis is a serious fungal infection formed as a result of improper neutrophilic response in the body. This is most commonly seen in immunocompromised children or in children with long term steroid usage, malnutrition and organ transplant recipients [8]. Patients with breaks in continuity of skin are at higher risk to develop cutaneous mucormycosis. Our patient had pressure necrosis due to ryles tube insertion in the right nostril. The germinated mucor spores can involve the adjacent tissue to cause necrosis but vascular invasion through cutaneous mucormycosis is less [9]. Very limited data was available explaining the pathophysiology of mucormycosis in MIS-C. The potential mechanisms that can be postulated in our case are shown in Figure 3. First, MIS-C is characterized by abnormalities of immune system leading to lower CD4+ and CD8+ T; elevated inflammatory cytokines interleukin (IL)-2R, IL-6, IL-10 and tumor necrosis factor alpha. Second, patients with severe MIS-C require longer duration of mechanical ventilation and prolonged intensive care unit stay that predispose to for hospital-infections and superadded fungal infections. Third, research data clearly demonstrate that individuals who have reduced phagocytes or have impaired phagocytic function are at higher risk to develop mucormycosis. For example, severely neutropenic patients are at greater risk for developing mucormycosis. Fourth, along with the host factors that predispose patients to Mucormycosis, mucorales possess virulence factors that enable the organism to cause disease.

Lastly' mucorales possess virulence factors that enable the organism to case disease. Lastly the occurrence of mucormycosis in this patient may be secondary to prolonged usage of systemic steroids which are known to be immunosuppressive agents. In a retrospective study of nine COVID-19 patients with aspergillosis, seven patients had received systemic steroids and five patients had received tocilizumab,z a IL-6- antagonist [10]. The use of glucocorticoids is a known risk factor for the development of mucormycosis. Glucocorticoid-induced immunosuppression, hyperglycemia and lymphopenia predispose these patients to the pathogenesis of mucormycosis [9]. Few other predisposing factors for the development of mucormycosis in COVID-19 patients are lympophenia and inflammation of endothelium secondary to viral infection. Extensive damage to the endothelium might facilitate easy adhesion and penetration of mucorales. These are very important initial steps in the etiopathogenesis of mucormycosis. There are four principles for managing mucormycosis to have better outcome: early diagnosis, elimination of the predisposing factors (if possible), surgical debridement of affected tissue and well-suited pharmacotherapy [11]. White *et al*. conducted a prospective study of 135 adults infected with COVID-19 and showed its high association with secondary fungal infection accounting the incidence to be 26.7 per [12]. Song *et al*. performed a study which focused on the association between COVID-19 and invasive fungal sinusitis and showed that there is an increased risk of developing invasive saprophytic infection in patient suffering or recovered from COVID-19 infection [13]. In a recent review, the incidence of secondary bacterial or fungal infection in individuals suffering from COVID-19 was recorded as 8 percent which was attributed to the longer duration of usage of broad spectrum antibiotics and systemic steroids [14]. A known case of diabetes developed rhino-orbital mucormycosis after prolonged therapy of corticosteroids during the management of COVID-19 [15]. Extensive spread of mucormycosis (involving lungs, hilar lymph nodes, brain and kidney) was found in a twenty-two-year-old COVID-19 patient during her postmortem examination [16]. According to data from a print media, a 15-year-old boy in Ahmedabad (Gujarat) had mucormycosis after post-COVID-19 recovery in which MIS-C can be considered as a possibility. The primary goals of treatment of a patient with MIS-C include reversal of shock and reversal of organ dysfunction and associated complications. Multiapproach management is recommended to treat MIS-C with mucormycosis including intensivists, immunologists, cardiologists, rheumatologists, and expert in infectious disease. Supportive care plays a major role in all cases; anti-inflammatory and immunomodulatory therapies are of utmost importance in severely ill patients. Early identification and intervention of shock and hypoxemia is linked with better outcomes in MIS-C patients. Corticosteroids are used along with IV immunoglobulin are used in patients in whom conventional treatment is not that fruitful. The usage of infliximab, anakinra, and tocilizumab has shown better disease course but data is sparse because of its easy availability. As MIS-C is a postinfectious inflammatory response, antiviral therapy does not prove to be beneficial. To the best of our knowledge, there are several cases of mucormycosis reported in cases with COVID-19 infection, but there is limited data available for the development of mucormycosis in MIS-C.

## Conclusion

Multisystem inflammatory syndrome (MIS-C) in Children is a multi-system inflammatory syndrome like condition that occurs post SARS-CoV-2 infection that is not self-limiting. It´s management mainly includes immunoglobulin infusion with steroid therapy. Our patient had received both immunoglobulin and steroid therapy. Following this, she developed mucormycosis. As there are negligible cases where patients developed mucormycosis post SARS-CoV-2 infection and none in pediatric age group, we came to the conclusion that this mucormycosis can be a result of the short steroid therapy given to our patient.
